# The AGE-RAGE Axis and Its Relationship to Markers of Cardiovascular Disease in Newly Diagnosed Diabetic Patients

**DOI:** 10.1371/journal.pone.0159175

**Published:** 2016-07-19

**Authors:** Ma. Etzabel Villegas-Rodríguez, Jaime Uribarri, Sergio E. Solorio-Meza, Martha E. Fajardo-Araujo, Weijing Cai, Sofía Torres-Graciano, Rubén Rangel-Salazar, Kazimierz Wrobel, Ma. Eugenia Garay-Sevilla

**Affiliations:** 1 Department of Medical Science. Division of Health Science. University of Guanajuato Campus. León, Guanajuato, México; 2 Department of Medicine. The Icahn School of Medicine at Mount Sinai, New York, New York, United States of America; 3 Mexican Institute of Social Security. León, Guanajuato, México; 4 Department of Chemistry, University of Guanajuato, Guanajuato, Guanajuato, México; University of Pittsburgh, UNITED STATES

## Abstract

**Aim:**

The purpose of the study was the simultaneous measurement of all the different components of the AGE-RAGE axis as well as several non-invasive markers of cardiovascular disease (CVD) in a cohort of newly diagnosed diabetic patients.

**Materials and Methods:**

In 80 newly diagnosed diabetic patients we measured serum carboxymethyllysine (CML), soluble RAGE (sRAGE) and peripheral mononuclear (PMNC) RAGE and AGER1 mRNA together with ICAM-1, VCAM-1, and malondialdehyde (MDA). We also assessed cardiovascular function by measurement of flow-mediated vasodilation (FMD), intima-media thickness (IMT) and arterial stiffness. Univariant correlation analysis was used to determine correlation between the variables in the study and multiple regression analysis was used to examine the association between the AGE-RAGE axis components and FMD, IMT and arterial stiffness.

**Results:**

Serum CML correlated positively with sRAGE, PMNC RAGE, HOMA-IR, ICAM-1, VCAM-1 and MDA, but inversely with PMNC AGER1. sRAGE and RAGE was positively correlated with AGER; IMT was positively correlated with HOMA-IR, ICAM-1, VCAM-1, MDA, and sRAGE and arterial stiffness had correlation with HOMA-IR, ICAM-1, VCAM-1, MDA, CML, sRAGE, AGER1 and RAGE. In multivariate analysis we found a significant relationship between CML with PMNC RAGE, HOMA-IR; sRAGE with VCAM-1 and MDA; PMNC RAGE with PMNC AGER1and CML; PMNC AGER1 with PMNC RAGE; FMD with sRAGE, CML and HbA1c; IMT with sRAGE, and arterial stiffness with sRAGE, sCML and AGER1

**Conclusions:**

We found significant and strong associations between the different components of the AGE-RAGE axis and also found significant association between AGE-RAGE axis markers, especially sRAGE with several noninvasive markers of cardiovascular disease risk. sRAGE, an easily measured parameter in blood, may potentially be used as a surrogate marker of AGEs-RAGE in patients with diabetes.

## Introduction

Worldwide there is an increasing prevalence of diabetes and its complications. Elevated levels of circulating advanced glycation end products (AGEs) are believed to play a major role in the pathogenesis of macrovascular and microvascular disease in diabetes mellitus [1]. Endogenous formation of AGEs is increased in diabetes as the result of hyperglycemia and increased oxidative stress in this condition. Recently, however, it has been demonstrated that food-derived AGEs play a major role in maintaining a high body pool of AGEs in diabetes [2].

AGEs interact with the receptor for AGEs (RAGE) on the cell membrane and induce deleterious effects via activation of nuclear factor κ-B, and increased oxidative stress and inflammatory mediators [3]. AGEs also combine with circulating soluble receptors for RAGE (sRAGE), C-truncated isoforms lacking cytosolic and transmembrane domains. sRAGE is formed from the cleavage of the native membrane receptor mediated by disintegrins and MMPs (matrix metal-lipoproteinase) [4] and circulates in the blood. Some studies have shown that sRAGE levels serve as a marker for development and progression of cardiovascular disease in diabetic and non-diabetic patients, but there is still no consensus whether it is high or low sRAGE that predicts worse cardiovascular outcome [5]. On the other hand, AGEs also react with AGER1, a receptor involved in the removal of AGEs, as well as in the maintenance of host defenses controlling their pro-inflammatory effects [6]. Decreased levels of AGER1 have been described in chronic diabetes and other conditions of sustained oxidative stress [7]. To date no study has simultaneously assessed these different components of the AGE-RAGE axis, namely serum AGEs, sRAGE, cellular RAGE and AGER1, in the same individual.

Cardiovascular disease (CVD) including atherosclerosis is a major cause of morbidity and mortality [8] in patients with diabetes. Atherosclerosis can be detected noninvasively in preclinical stages by measuring carotid intima media thickness (IMT) that permits detection of early structural changes in the vascular wall [9] and by flow-mediated vasodilation, a commonly used method to measure endothelial dysfunction [10]. Vascular endothelial damage is the first manifestation of atherosclerosis [11] and elevated concentration of adhesion molecules such as Vascular cell adhesion molecule-1 (VCAM-1) and Intercellular Adhesion Molecule-1 (ICAM-1) may be useful indicators of the development of atherosclerotic plaques at an early phase in diabetes [11]. Aortic stiffness is another non-invasive test that increases with age [12] and has been associated with an increased risk of cardiovascular events in the general population and in diabetic patients [13]. In the current study we measured simultaneously the different components of the AGE-RAGE axis as well as several non-invasive markers of CVD in a cohort of newly diagnosed diabetic patients. This allowed us to establish the relationship of these different components of the AGE-RAGE axis between themselves and with CVD markers.

## Materials and Methods

The study was performed on 80 newly diagnosed diabetic patients. These patients were recruited from a large community in Guanajuato, Mexico based on the results from an oral glucose tolerance screening test according to the American Diabetes Association (ADA) criteria. Inclusion criteria for participation in the screening test included: age 30–65 years, absence of active infection and no liver, cardiovascular or renal diseases. The institutional ethical committee at the University of Guanajuato approved the protocol and all subjects signed a consent form. At the initial study visit clinical and anthropometric evaluations were performed, a sample of fasting blood was obtained and non-invasive tests of cardiovascular status (see below) were assessed.

Plasma was processed the same day and used for measurement of glucose using glucose GOD-PAP (Lakeside, Mexico City), triglyceride, total cholesterol and HDL cholesterol using enzymatic colorimetric kits (Spinreact, Spain), creatinine and HbA1c by inmunoturbidimetry (Spinreact, Spain). Serum aliquots were stored at -80°C until further determination of the following biomarkers: insulin, measured by IRMA (Cisbio Bioassays); ICAM-1 (208±4.7 ng/mL) and VCAM-1(557±139.6 ng/mL) by ELISA (R&D Systems) with reference values according to the manufacturer, malondialdehyde (MDA) by ELISA (Abcam® Lipid Peroxidation (MDA) Assay) (0.95±0.71 nmol/ml values reported in healthy subjects) [14]; sRAGE by ELISA (R&D system) (443±187.8pg/mL unpublished values in healthy subjects in our group); ^ε^N-carboxymethyl-lysine (CML) in serum by ELISA (6.2±1.6 U/mL unpublished values in healthy subjects in our group), using non cross-reactive monoclonal antibodies (4G9) raised against synthetic standard, CML-BSA as previously described [15].

### RAGE and AGER1 mRNA measurement

#### Peripheral blood mononuclear cells (PMNCs)

PMNCs were separated from fasting, EDTA anticoagulated blood by Ficoll-Hypaque Plus gradient (Amersham Biosciences, Uppsala, Sweden) and used to isolate mRNA. Total RNA was extracted by Trizol (Molecular Probes, Inc., Eugene, OR). The extracted RNA had an OD 280:260 ratio between 1.8 and 2.0. Total RNA was reversed transcribed using Superscript III RT (Invitrogen, Carlsbad, CA).

#### Polymerase chain reaction (PCR) assay

Quantitative SYBR Green real-time (Roche, IN, U.S.A.) was performed to analyze expression of mRNA for AGER1 and RAGE. Briefly, 7.5 μl of template cDNA were added to a final volume of 20 μl containing 1X SYBR Green PCR master mix and 5 pM of the primers. Amplification was performed with 40 cycles of denaturation at 95 C for 15 sec, annealing at 52 C for 20 sec, and elongation at 72 C for 30 sec. Sequences of the primers used for real-time PCR were: AGER1, forward primer, 5’-CTGGGGCTCTTCATCTTCAG-3’; reverse primer 5’-GTTGCATCTCCCACAGAGGT-3’; RAGE, forward primer, 5’-AGGAGCGTGCAGAACTGAAT-3’; reverse primer 5’-TTGGCAAGGTGGGGTTATAC-3’. The mRNA levels were acquired from the value of threshold cycle (C_T_) of AGER1 and RAGE and normalized against C_T_ of glyceraldehyde-3-phosphate dehydrogenase (GAPDH) [16].

### Non-invasive Cardiovascular Assessment

#### Assessment of brachial artery flow-mediated vasodilation (FMD)

A pneumatic cuff was placed on the forearm, distal to the ultrasound image site and was inflated to above systolic pressure for 5 min to induce ischemia. On deflation of the cuff, the increased flow results in shear stress, which activates endothelial NO synthase to release NO via the L-arginine pathway. The NO diffuses to the smooth muscle cells, causing them to relax, resulting in vasodilation. Ultrasound images of the brachial artery were obtained at baseline and after release of the cuff. FMD was measured as the percentage change in brachial artery diameter from baseline to the maximum increase in diameter. An increase less than 10% in the diameter of the artery suggests the presence of arterial disease [17].

#### Carotid intima-media thickness (IMT)

Carotid ultrasound was performed with subjects resting in supine position with slight hyperextension of the neck. The intima-media thickness (IMT) is the double-line parallel pattern in the carotid district that is commonly visualized by the B-mode vascular ultrasound by longitudinal scanning. IMT is defined as the distance between the luminal–intimal interface and the media–adventitial interface of the common carotid artery. A value of IMT ≥ 0.84 mm indicates the presence of cardiovascular disease [18]. The assessments were made using a ACUSON X150 ultrasound machine (Siemens, Mexico City, Mexico).

#### Arterial Stiffness index

Stiffness index was calculated according to Mackenzie’s formula [19]. According to this formula, stiffness index = ratio of ln(systolic/diastolic pressures) to (relative change in diameter): β = [ln(Ps/Pd)]/[(Ds-Dd)/Dd] (P, pressure; D, diameter; s, systolic; d, diastolic). The blood pressure was measured with a calibrated electronic sphygmomanometer and vascular diameter by two-dimensional ultrasound.

### Statistical Analysis

Results are expressed as mean ± SD for continuous variables. Pearson’ correlation analysis was used to determine univariant correlation between the different variables in the study. Multiple regression analysis was used to examine the association between FMD, IMT and arterial stiffness and also sCML, sRAGE, expression of RAGE and AGER1 with all the other significant variables found by correlation analysis. All analyses were performed using Statistica 7 software (Statsoft Inc., Tulsa, OK). Significance was defined as a value of p <0.05.

## Results

From February 2013 to May 2014 in the city of Leon, Guanajuato, Mexico, we performed oral glucose tolerance tests in 660 adults between the ages of 35 and 60 years old with no prior history of diabetes. Based on the ADA criteria 80 of these subjects were diagnosed with diabetes mellitus and asked to participate in our study.

The study included these 80 patients newly diagnosed with diabetes mellitus type 2. None of these patients were taking diabetic medications at the time of the study. Clinical and metabolic characteristics of the group are shown in Table 1. 55 (69%) were women and 25 (31%) men, age 48.5±7.3, BMI of 29.9±6.6, normal blood pressure and normal renal function as defined by serum creatinine. As expected in a diabetic population, fasting blood glucose and HbA1c levels were elevated. Values for ICAM-1 and VCAM-1 were higher than the values reported in healthy subjects; values for sCML and sRAGE were higher than values in historic controls in our group. IMT, FMD were higher than the values reported in healthy subjects [20] and arterial stiffness values were within normal range.

**Table 1:** Clinical and metabolic features of the patients.

Demographic and anthropometric variables	Study subjects	Healthy subjects*
Women /Men (n)	55/25	
Age (years)	48.5±7.3	
Weight (kg)	76.6±17.1	
Height (m)	1.6±0.01	
BMI (Kg/m^2^)	29.9±6.6	
Sistolic blood pressure (mmHg)	121.8±6.5	
Diastolic blood pressure (mmHg)	76.3±6.7	
**Biochemical variables**		
Glucose (mmol/L)	7.9±2.4	
HbA1c (%)	6.5±1.1	
HbA1c (mmol/mol)	48±7	
Creatinine (μmol/L)	76.9±10.6	
Triglycerides (mmol/L)	2.1±0.8	
Cholesterol (mmol/L)	5.1±1.0	
HDL-C (mmol/L)	1.5±0.2	
LDL-C (mmol/L)	2.6±0.8	
VLDL-C (mmol/L)	1.0±0.4	
Insulin (uUI/mL)	8.3±3.2	
HOMA- IR	3.0±1.4	
ICAM-1 (ng/mL)	251.5±59.4	208±4.7 ng/mL #
VCAM-1 (ng/mL)	694.2±163.6	557±139.6 ng/mL #
MDA (nmol/mL)	3.1±2.2	0.95±0.71 [14]
CML (U/mL)	14.1±4.2	6.2±1.6 U/mL§
sRAGE (pg/mL)	1027.7±416.3	443±187.8pg/mL §
**Receptor expression**		
Expression of RAGE (UA)	31.1±3.5	
Expression of AGER1 (UA)	25.7±0.9	
**Cardiovascular Risk Assessments**		
IMT (mm) _intima-media thickness_	0.8±0.2	
FMD (%) _flow-mediated vasodilation_	16.2±7.1	
Arterial Stiffness	3.7±2.3	

All values are expressed as mean ± SD.

*Values in healthy subjects were obtained either from reference values provided by the kit manufacturer # or represent unpublished values in healthy adult subjects in our laboratory§

Correlations between the different variables within the AGE-RAGE axis

**Fig 1:**
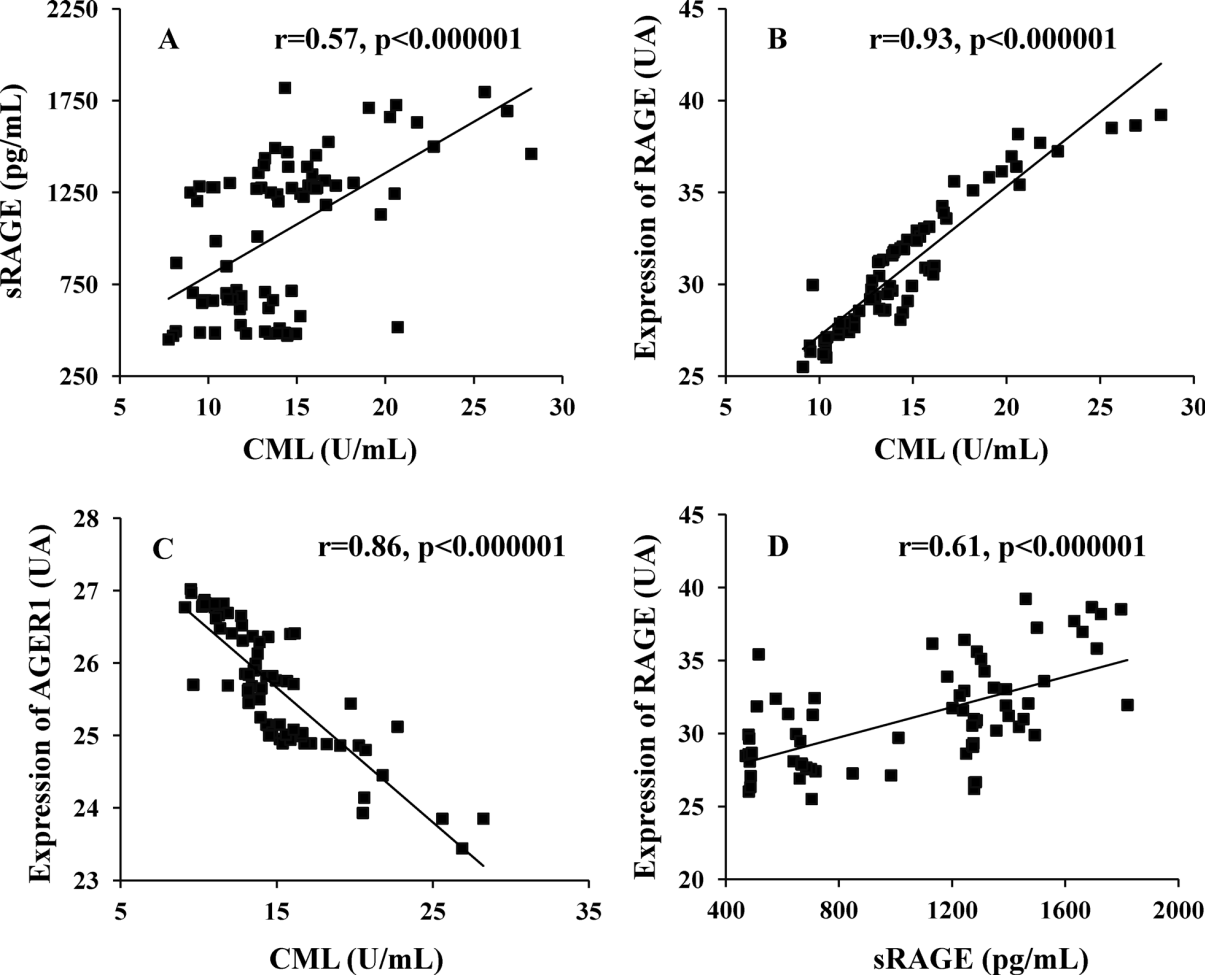
(A) Show the correlation between sRAGE with CML. (B) Expression of RAGE with CML. (C) Expression of AGER1 with CML and (D) Expression of RAGE with sRAGE

Table 2 and Fig 1 show significant positive correlations of serum CML with sRAGE and PMNC RAGE, but inversely with AGER1. sRAGE was positively correlated with sCML and PMNC RAGE and negatively with PMNC AGER1.

**Table 2:** Relationships among CML, sRAGE, Expression RAGE and AGER1, insulin resistance, oxidative stress and endothelial damage.

Variable	CML r	sRAGE r	Expression RAGE r	Expression AGER1 r
Age (years)	-.205	.109	-.129	.089
BMI (Kg/m^2^)	.308^2^	.328^2^	.646^3^	-.694^3^
HbA1c (%)	.230^1^	.096	.200	-.084
HOMA-IR	.599^3^	.682^3^	.557^3^	-.455^3^
ICAM-1 (ng/mL)	.638^3^	.888^3^	.654^3^	-.615^3^
VCAM-1 (ng/mL)	.585^3^	.914^3^	.611^3^	-.572^3^
MDA (nmol/mL)	.611^3^	.909^3^	.643^3^	-.609^3^
CML (U/mL)		.566^3^	.931^3^	-.861^3^
sRAGE (pg/mL)	.566^3^		.614^3^	-.571^3^
Expression of RAGE (UA)	.930^3^	.609^3^		-.920^3^
Expression of AGER1 (UA)	-.857^3^	-.571^3^	-.920^3^	

^1^p<0.01

^2^p<0.001

^3^p<0.0001

Correlations between the variables within the AGE-RAGE axis and markers of glucose metabolism, oxidative stress and endothelial dysfunction markers

Table 2 shows that sCML correlated positively with BMI, HbA1c, insulin resistance index (HOMA-IR), ICAM-1, VCAM-1 and MDA. sRAGE also correlated positively with BMI, HOMA-IR, ICAM-1, VCAM-1, and MDA.

PMNC RAGE was positively correlated with BMI, HOMA-IR, ICAM-1, VCAM-1, and MDA, while PMNC AGER1 was negatively correlated with BMI, HOMA-IR, ICAM-1, VCAM-1, and MDA. We performed multivariate regression analysis of each of the variables within the AGE-RAGE axis as a dependent variable and all variables that showed statistical significance in correlation analysis as independent variables and we found a significant relationship between CML with PMNC RAGE (R^2^ = 0.87, p<0.00001), HOMA-IR (R^2^ = 0.55, p<0.02) and BMI (R^2^ = 0.35, p<0.001); sRAGE with VCAM-1 (R^2^ = 0.95, p<0.01), MDA (R^2^ = 0.98, p<0.01), and marginally with ICAM-1(R^2^ = 0.9, p<0.048); PMNC RAGE with PMNC AGER1(R^2^ = 0.73, p<0.000001) and CML (R^2^ = 0.71, p<0.00001); PMNC AGER1 with PMNC RAGE (R^2^ = 0.79, p<0.000001) and BMI (R^2^ = 0.37, p<0.01).

**Fig 2:**
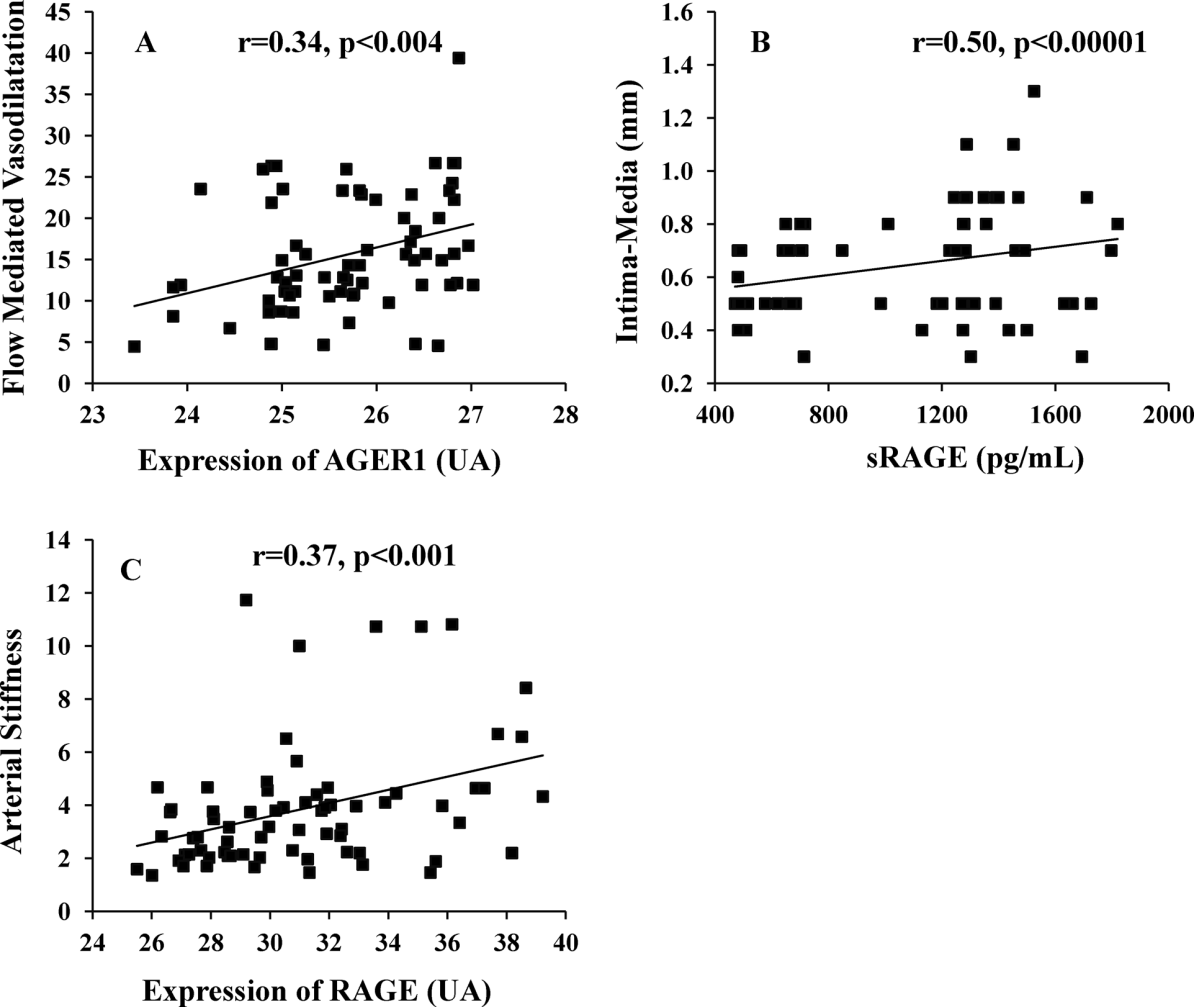
(A) Show the correlation between flow-mediated vasodilation with expression of AGER1. (B) Intima-media thickness with sRAGE and (C) Arterial Stiffness with Expression of RAGE.

Correlation between the AGE-RAGE axis variables and non-invasive Cardiovascular Assessment FMD was negatively correlated with HbA1c, HOMA-IR, ICAM-1, VCAM-1, MDA, CML, sRAGE and PMNC RAGE and was positively correlated with PMNC AGER1 (Table 3 and Fig 2A). IMT was positively correlated with HOMA-IR, ICAM-1, VCAM-1, MDA, and sRAGE (Table 3 and Fig 2B). Arterial Stiffness correlated positively with HOMA-IR, ICAM-1, VCAM-1, MDA, CML, sRAGE, PMNC RAGE and PMNC AGER1 (Table 3 and Fig 2C).

**Table 3:** Relationships among flow-mediated vasodilation, intima-media thickness_,_ Arterial Stiffness, and variables in the study.

Variable	FMD r	IMT r	Arterial Stiffness r
Age (years)	-.146	.123	.178
BMI (Kg/m^2^)	-.126	.010	.087
HbA1c (%)	-.307^1^	-.069	.139
HOMA-IR	-.465^3^	.329^2^	.310^2^
ICAM-1 (ng/mL)	-.479^3^	.466^3^	.407^3^
VCAM-1 (ng/mL)	-.463^3^	.474^3^	.392 ^3^
MDA (nmol/mL)	-.462^3^	.475^3^	.400^3^
CML (U/mL)	-.461^3^	.159	.424^3^
sRAGE (pg/mL)	-.541^3^	.500^3^	.483^3^
Expression of RAGE (UA)	-.395^2^	.127	.368^2^
Expression of AGER1 (UA)	.341^1^	-.226	.261^1^

^1^p<0.01

^2^p<0.001

^3^p<0.0001

When we performed multivariate regression analysis of each of the cardiovascular assessment parameters as a dependent variable and adding all variables that showed statistical significance in correlation analysis as independent variables we found a significant relationship between FMD, with sRAGE (R^2^ = 0.6, p<0.0001), CML (R^2^ = 0.88,p<0.007), HbA1c (R^2^ = 0. 47, p<0.008) and PMNC RAGE (R^2^ = 0.88,p<0.03). IMT, correlated with sRAGE (R^2^ = 0.85,p<0.01); Arterial Stiffness, with sRAGE (R^2^ = 0.84, p<0.001), sCML (R^2^ = 0.79p<0.0003), and PMNC AGER1, (R^2^ = 0.77, p<0.003) (Table 4). These relationships did not change significantly when adjusting for BMI.

**Table 4:** Multivariate analysis of relationships between vascular function and significantly variables in correlation analysis.

Variable	FMD		IMT		Arterial Stiffness	
	β	P value	β	p value	β	P value
sRAGE (pg/mL)	-0.650	0.0001	0.389	0.007	0.842	0.001
CML (U/mL)	-0.754	0.007			0.818	0.0003
HbA1c (%)	-0.355	0.008				
Expression of RAGE	0.614	0.03				
Expression of AGER1					0.621	0.003

## Discussion

In this population of patients with newly diagnosed diabetes type 2 we found significant associations between the different components of the AGE-RAGE axis. sCML was associated positively with RAGE and sRAGE and inversely with AGER1, while RAGE was associated positively with sRAGE and inversely with AGER1. We also found significant associations between AGE-RAGE axis markers, especially sRAGE, and several noninvasive markers of CVD risk including flow-mediated vasodilation, intima-media thickness and arterial stiffness.

Diabetic patients are known to have increased levels of AGEs in the circulation and RAGE in their mononuclear cells [21]. AGEs stimulate RAGE and RAGE activation seems to mediate most of the biological effects of AGEs by generating reactive oxygen species *(*ROS) and stimulating inflammatory pathways. In the diabetic state, endogenous AGE production is increased and RAGE is up-regulated, whereas the AGEs scavenger receptor, namely AGER1, is down-regulated, possibly resulting in lower AGEs clearance [21]. All of these findings suggest that diabetes triggers AGE-mediated mechanisms, which could contribute to the increased cardiovascular morbidity and other diabetes-related complications. [22]. Endothelial dysfunction is diagnosed using physical and biochemical methods. Flow-mediated vasodilation is the most widely used and the most sensitive among the physical methods [23], while biochemical detection of endothelial dysfunction is frequently performed measuring markers such as VCAM-I and ICAM-I among others [24]. In this study, markers of endothelial dysfunction and oxidative stress (OS) were also associated with sCML, sRAGE and PMNC RAGE.

Some investigators have already shown the relationships among serum levels of CML and RAGE and endothelial function in established diabetic and nondiabetic subjects [25, 26], supporting our findings in this group of patients with newly diagnosed diabetes.

We found a positive association of CML with sRAGE, as previously reported in diabetic patients and non-diabetic patients [27]. There is an extensive literature on sRAGE and its use as a biomarker of CVD risk, but with variable directions [5, 28]. On the other hand, our data support the concept that in diabetic patients, AGE stress increases RAGE and sRAGE. Inferring a direct role for increased sRAGE in the development of cardiovascular disease is beyond the scope of this study.

Expression of AGER1 mRNA has been found to be directly correlated with sCML in healthy subjects [29], but it was suppressed in states of sustained high sCML such as diabetes [21]. Our findings showed a negative correlation between AGER1 and sCML in a population of newly diagnosed diabetic patients with high oxidative stress and inflammation supporting previous findings in the literature [21]. We found that PMNC AGER1 was negatively associated with sRAGE, PMNC RAGE and all other markers of OS and endothelial dysfunction. Of interest, in vitro data suggests that overexpression of AGER1 suppresses RAGE pro-inflammatory signaling pathways, and contributes to maintain AGE homeostasis [30].

Our finding of a positive association between PMNC RAGE and sRAGE contrasts previous findings by Tam XH and et al [4] who found a weak inverse correlation between these two variables. It is possible that the inadequate glycemic control in Tam’s study patients may explain the difference with our study.

We found a significant and positive association of CML with HOMA-IR, an index of insulin resistance. This has been frequently reported in the literature [31]. Moreover, interventions aimed at decreasing sCML have also produced improvement in the HOMA-IR [21].

Our finding of a positive association between sRAGE and HOMA-IR contradicts a recent study [32] in which no statistical association of sRAGE with IR was found in people with newly diagnosed type 2 DM. We are not sure why the different findings, but it is worth mentioning that the mean of their sRAGE values was much lower (646pg/mL) than ours.

We found a positive correlation of sRAGE with malondialdehyde, a marker of OS. This fits the well-established AGE-induced RAGE activation that promotes the activity of the nuclear factor –κB resulting in increased expression of cytokines, adhesion molecules and induction of OS [33].

The concept that sRAGE levels may be elevated in response to serum AGE levels and reflect tissue RAGE expression in diabetes is supported by similar elevations in levels of ICAM-1 and VCAM-1 under the same circumstances since RAGE belongs to the same immunoglobulin superfamily as ICAM-1 and VCAM-1 [34]. It has also been suggested that sRAGE can be a biomarker for RAGE-mediated disease, especially vascular disease [35].

Our results show positive associations of sCML, sRAGE and PMNC RAGE with arterial stiffness, while sRAGE also had a positive association with IMT and negatively with FMD. Recently di Pino A and et al [36] also found positive association between IMT with sRAGE in simple and multiple regression analysis suggesting that sRAGE may be a good marker of cardiovascular risk profile. Kajikawa M and et al [37] showed negative association of FMD and sCML similar to our results. We measured IMT and arterial stiffnes (AS) by ultrasound, whose reproducibility may be affected by multiple factors [38], and therefore we also measured biochemical parameters to evaluate the cardiovascular risk and support our results.

Strengths of the study include the homogeneity of the study participants, their recent diagnosis of diabetes and the lack of use of medications for diabetes. A weakness of the study is the lack of a control group to clearly define reference values since the population studied comes from a specific region in Mexico and therefore the findings might not be generalizable. Similar findings reported in the international literature, however, support our results. We had only one value measured in each patient and therefore we cannot prove, only suggest, the use of these markers as predictors of risk.

## Conclusion

Our results show a significantly and strong association between the different components of the AGE-RAGE axis and also found significant association between AGE-RAGE axis markers, especially sRAGE, with several noninvasive markers of cardiovascular disease risk. These findings support the use of sRAGE, a simple assay, as a biomarker of AGE-RAGE activity in patients with diabetes.
